# Attentional bias modification in depression through gaze contingencies and regulatory control using a new eye-tracking intervention paradigm: study protocol for a placebo-controlled trial

**DOI:** 10.1186/s12888-016-1150-9

**Published:** 2016-12-08

**Authors:** Carmelo Vazquez, Ivan Blanco, Alvaro Sanchez, Richard J. McNally

**Affiliations:** 1 Faculty of Psychology, Complutense University at Madrid, 28223 Madrid, Spain; 2 Department of Experimental Clinical and Health Psychology, Ghent University, Henri Dunantlaan 2, B-9000 Ghent, Belgium; 3 Department of Psychology, Harvard University, 33 Kirkland Street, Cambridge, MA 02138 USA

**Keywords:** Depression, Cognitive processes, Attentional biases, Attentional bias modification, Eye-tracking, Cognitive bias modification, Dysphoria

## Abstract

**Background:**

Attentional biases, namely difficulties both to disengage attention from negative information and to maintain it on positive information, play an important role in the onset and maintenance of the disorder. Recently, researchers have developed specific attentional bias modification (ABM) techniques aimed to modify these maladaptive attentional patterns. However, the application of current ABM procedures has yielded, so far, scarce results in depression due, in part, to some methodological shortcomings.

The aim of our protocol is the application of a new ABM technique, based on eye-tracker technology, designed to objectively train the specific attentional components involved in depression and, eventually, to reduce depressive symptoms.

**Methods:**

Based on sample size calculations, 32 dysphoric (BDI ≥13) participants will be allocated to either an active attentional bias training group or a yoked-control group. Attentional training will be individually administered on two sessions in two consecutive days at the lab. In the training task series of pairs of faces (i.e. neutral vs. sad; neutral vs. happy; happy vs. sad) will be displayed. Participants in the training group will be asked to localize as quickly as possible the most positive face of the pair (e.g., the neutral face in neutral vs. sad trials) and maintain their gaze on it for 750 ms or 1500 ms, in two different blocks, to advance to the next trial. Participants’ maintenance of gaze will be measured by an eye-tracking apparatus. Participants in the yoked-control group will be exposed to the same stimuli and the same average amount of time than the experimental participants but without any instruction to maintain their gaze or any feedback on their performance. Pre and post training measures will be obtained to assess cognitive and emotional changes after the training.

**Discussion:**

The findings from this research will provide a proof-of-principle of the efficacy of eye-tracking paradigms to modify attentional biases and, consequently, to improve depressed mood. If the findings are positive, this new training approach may result in the improvement of cognitive bias modification procedures in depression.

**Trial registration:**

This trial was retrospectively registered on July 28, 2016 with the ClinicalTrials.gov NCT02847793 registration number and the title ‘Attentional Bias Modification Through Eye-tracker Methodology (ABMET)’.

**Electronic supplementary material:**

The online version of this article (doi:10.1186/s12888-016-1150-9) contains supplementary material, which is available to authorized users.

## Background

Depression is a very common form of psychopathology associated with adverse personal (individual suffering, suicide) and societal costs (sick leave, treatment costs) –[1]. Despite the wide range of pharmacological and psychological interventions available, existing treatments for depression have shown limited efficacy and unwanted side effects [2, 3]. Furthermore, although some of the current interventions are efficacious in the short term, relapse is common after first episodes of depression resolve. At least 50% of those who recover from a first episode of depression experience at least one subsequent episode [4]. Therefore, clinical researchers have endeavored to identify core mechanisms involved in the development and maintenance of depression as potential targets for novel interventions.

### Identifying evidence-based targets for intervention

In contrast to healthy individuals, who are characterized by a preferential processing of positive information, depressed individuals exhibit cognitive biases such as sustained attention to negative information [5], a bias for interpreting ambiguous information in a negative fashion [6], and a bias for preferentially retrieving negative material from both implicit [7] and explicit memory [8].

These information processing biases appear closely interrelated. For instance, it has been proposed that depression and risk for depression are associated with a vicious cycle whereby negative mood states are maintained by sustained attention to negative information [9], which activates elaboration mechanisms and negative self-schemas [10]. As this pattern becomes habitual, chronic low mood is maintained. Thus, current views of depression emphasize the role of cognitive, especially attentional, mechanisms in the etiology and maintenance of depression [11]. First, attentional biases for negative information precede subsequent elaboration of such information, thereby fostering interpretation or memory biases [12, 13]. Second, experimental manipulation of early-stage attentional biases leads to differential patterns of elaborative processing [14] that ultimately influence emotional regulation [15]. Therefore, therapeutic correction of attention biases should have beneficial effects in depression.

Researchers have developed increasingly refined methods for isolating the pathogenic role of attentional biases in depression such as eye-tracking procedures and appropriate specific mood-congruent stimuli [16]. These new procedures have revealed that depression is characterized by sustained attention to negative information rather than rapid orienting to such information [16–19]. Such attentional bias occurs for both pictorial emotional stimuli [18, 20] and emotional words [21], and is related to maladaptive forms of emotional regulation in depression, such as increased rumination [22, 23] and a reduced use of reappraisal strategies [24, 25]. Importantly, attention biases in depression are not restricted to the processing of negative information. In contrast to healthy people [26, 27], depressed participants also show a decreased attention to positive information [28, 29]. There is meta-analytic evidence showing that people with depression exhibit slower engagement and faster disengagement from positive information [16].

In sum, depressed individuals seem characterized by a double bias in their deployment of attention (i.e., sustained attention to negative stimuli and reduced attentional engagement and maintenance of attention to positive stimuli) [30]. If such attention biases play a casual role in the etiology or maintenance of depression, then their correction should facilitate recovery from the disorder.

### Current procedures to correct biased cognitive processes in depression

A new set of cognitive techniques, known as Cognitive Bias Modification (CBM), has been developed to modify cognitive biases in emotional disorders. In a seminal experiment, healthy participants were randomly assigned to receive training designed to direct their attention either toward or away from threat-related words [31]. The training procedure was an adaptation of the dot-probe paradigm for measuring attentional bias for threat [32]. In the original paradigm, a pair of words (e.g., one neutral and the other threat-related) appeared on a computer, one above center screen and one below center screen. After 500 ms, the word pair vanished and a dot appeared in the location vacated by one of the words. Participants were told to push a button to indicate the dot’s position as soon as they detected the dot. A quicker response to the dot when it occurs in the previous location of a threatening stimulus is interpreted as vigilance for threat. Authors modified this paradigm to train attention away from threat by having the dot repeatedly replacing neutral words and toward threat by having it repeatedly replacing threatening words. Then participants were exposed to a laboratory stressor. It was found that the group trained to attend to neutral stimuli was significantly less emotionally reactive to the stressor than was the group trained to threat stimuli. The researchers concluded that attentional bias for threat heightens anxiety proneness, and that reducing such bias diminishes anxiety proneness. This experiment inspired the development of Attention Bias Modification (ABM) procedures as well as indicated the causal effects of cognitive processes on emotion [33, 34].

Because of its conceptual appeal (i.e., the possibility of changing emotions through pure cognitive interventions), its reduced costs and its acceptability, ABM procedures have been proposed as valuable clinical tools [34, 35]. Yet, only a few ABM studies have been conducted in depression so far and, with rare exceptions [36, 37], most involved healthy participants scoring high on questionnaire measures of depression [38–42]. Moreover, literature reviews of ABM efficacy in emotional disorders have found inconsistent results. Some meta-analyses have found moderate or large effects for change in attention bias after ABM [43, 44], whereas others have found small effect sizes [45, 46]. Similar inconsistency has been found for symptom improvements after training. Medium effect sizes have been found in some meta-analyses [44], whereas small effects sizes were found in others [43, 47]. In a more refined meta-analysis, which specifically focused on ABM in distinct clinical conditions (i.e., anxiety, depression, and substance abuse), the authors concluded that attentional biases and symptom changes were successfully reduced in anxiety (both in clinical and healthy samples) although the average effect size was small. Yet, the authors did not find significant changes for other clinical conditions, such as depression [46]. A similar negative conclusion was reached in a recent meta-analysis on CBM procedures in anxiety and depression [45]. Although this meta-analysis did not separate the results by different types of procedures (e.g., interpretation bias training as opposite to ABM), as in the Mogoase et al.’s meta-analysis [46], their conclusions were rather pessimistic on the utility of CBM procedures. In the case of depression, the moderate significant effect sizes found when comparing normal controls to subclinical samples (g = 0.43) or clinical samples (g = 0.33) disappeared when outliers and publication bias were considered. The authors of this meta-analysis concluded that CBM is not as promising as many had hoped [34].

### Conceptual and methodological limitations of current ABM procedures

One possible explanation for discrepancies in the literature of ABM, and the modest effect sizes it has produced, is that current procedures to correct biases are suboptimal. Cristea et al.’s meta-analysis [45] concluded that the procedural diversity of CBM studies may render meta-analysis premature. They recommended that researchers should focus on developing high quality trials sufficiently powered. Yet it is also plausible that limitations of current methods, such as unreliability of the dot-probe paradigm or the failure of researchers to distinguish between different aspects of attention (e.g., engagement, disengagement) means that there is still some room for significant methodological and conceptual improvements in current suboptimal ABM procedures [48]. ABM therapeutics should be rooted in advancements in both basic and clinical science, and should target specific processes (e.g., gaze behavior) associated with specific attention biases characterizing each type of disorder. More specifically, new ABM methods should address the issues described below.

- *Trial-by-trial feedback.* Most of the extant ABM tasks, such as the modified dot-probe task, are very repetitive [46]. These procedures are based on a model of automatization as a way to modify cognition [49]. ABM training typically consists of hundreds [39, 41], or thousands [37, 42], of repetitive trials taking the procedure from one single session [38, 39] to several weeks [36, 41, 42]. ABM is accordingly very tedious which may impair the efficacy of cognitive training. In fact, patients report that ABM is less interesting than other CBM procedures such as interpretation bias modification training [43]. Improvements in ABM procedures that render them more motivationally engaging for patients, such as performance feedback and reward, are desirable as such changes may foster more robust changes [50]. In fact, anticipation of reward or receipt of reward upon performance shapes visual selective attention strategies [51]. Thus, one possible way of optimizing participants’ attentional performance in ABM paradigms could be providing trial-by-trial feedback on performance [52]. For example, in a recent study testing ABM through reward [53], the authors used a dot-probe paradigm with disgust and neutral faces in a sample of clinically anxious participants. For each trial, participants received an auditory signal following correct responses plus feedback on how much money they were earning. The results showed that the six-session ABM training significantly improved the participants’ attention towards neutral faces, although it did not reduce anxiety symptoms.
- *Targets of attentional training.* Given the double attentional bias in depression [30, 54], ABM should target both biases, increasing attention to positive stimuli while reducing attention to negative stimuli. Moreover, it should be directed toward modifying later components of attention as these are the most affected in depression [16, 18]. That is, ABM should be aimed at reducing the time to disengage from negative information, and increasing the maintenance of attention to positive information once attention has been captured by these features.
- *Training procedure: eye tracking methodology.* Serious concerns about the reliability of the dot-probe paradigm [55] may render it unsuitable for ABM except under specific conditions [56, 57]. Even more importantly, the dot probe task does not allow the identification and differentiation of precise time-based components of attentional performance (i.e., attentional engagement, maintenance, and disengagement) [58] that are especially relevant for depression [16]. Therefore, eye-tracking paradigms may serve not only to distinguish relevant attention processing stages but also to adequately target and modify them.
- *Assessment of transfer effects to emotional functioning and symptoms.* Another important methodological consideration refers to the detection of transfer effects after receiving ABM training. It is possible that outcomes of ABM procedures are not immediately detectable or they appear only under specific circumstances. A study using dot-probe training with either positive faces or positive words [37] found that the face ABM modality was ineffective in reducing depressive symptoms in patients whose depression had remitted at the time of training but after 4 weeks participants showed a significant symptom reduction. The authors interpreted their results in terms of the possibility that ABM training might have reduced the vulnerability to develop future episodes of depression. These results are in line with the suggestion that emotional changes resulting from ABM would only be visible after a period of time has passed or in response to a subsequent stressor [41]. Thus, it might be possible that the lack of significant transfer effects in depressive symptom reduction in previous ABM studies [36, 39] would be, at least in part, due to limitations in assessment procedures. Future ABM procedures require the evaluation of transfer effects to other tasks (e.g., stress tests).
- *Control group.* It is difficult to find a conceptually valid control group for CBM paradigms [59]. For instance, in the typical dot-probe control (placebo) condition, the dot follows the neutral stimulus in half of the trials and follows the negative stimulus in the remaining trials. Hence, there is no contingency between stimulus valence and the dot probe. Although this procedure is routine in experimental psychology [60], the rationale for using it in ABM is debatable. In dot-probe based ABM procedures, the control group is not a pure control condition since participants are exposed to threat stimuli contingencies (as well as neutral stimuli contingencies), in half of the trials.

### The proposal of a new ABM intervention

Taken together, these conceptual and methodological considerations point towards modifications in current ABM procedures [61]. The aim of this study will be to apply eye-tracking methods to modify specific components of attentional bias in depression. Eye-tracking technology enables us to train attention by following strict performance and time-based criteria as well as to specify the components of attention (i.e., disengagement from negative information, engagement and maintenance in positive information) to be targeted in the training, critical to providing a theory-driven intervention [59]. The proposed ABM procedure accomplishes this by providing individualized trial-by-trial feedback on trainees’ gaze allocation during the attentional processing of different emotional stimuli. This new procedure should maximize awareness of attention allocation to increase regulatory control in redirecting attention in an adaptive manner [15, 62].

The inclusion of individualized feedback on trainees’ performance during the attention training [57], in combination with explicit guidance of attention in the intended direction [15], may be a promising approach. Explicitly instructing participants to redirect attention from negative to positive information and providing them with online feedback on their performance can increase top-down regulation of attention and diminish emotional reactions to stressors [15]. Therefore, in the current trial, we aimed to develop a novel ABM method for treating depression-related attention biases, relying not only on the use of eye-tracking contingencies, but also on online feedback and explicit implementation of top-down regulation to train attention patterns. Furthermore, a significant improvement of our proposed experimental design over previous studies is related to the control group. In our study we will design a ‘yoked-control’ condition where participants in the control group will be exposed to the same experimental stimuli the same amount of time than participants on experimental condition, but without receiving any type of training or contingency for those stimuli.

In contrast to other recent innovative approaches [15, 57], our trial counts as a proof of principle illustrating how researchers can use eye-tracking methods to modify gaze patterns in subclinical depression. Furthermore, we aim to test whether training effects transfer to measures of emotional responding under non-stressful and stressful conditions and whether beneficial effects are evident on other measures relevant to emotional regulation (i.e., emotional identification, interpretation biases).

## Methods/Design

### Study design overview

The design will involve repeated-measures across two assessment times (pre-attentional training and post-attentional training). The pre-post assessment will include questionnaires evaluating depression and anxiety symptoms as well as psychological vulnerability factors for depression (i.e., rumination, thought suppression, and approach and avoidance behavior) and protective factors against depression (i.e., satisfaction with life, optimism and well-being).

The pre-post assessment will include the evaluation of direct effects of the training on attentional performance by using a visual attention task, Attentional Bias Assessment (ABA), different from the one employed in the (Attentional Bias Modification Training, ABM-T). Furthermore, the pre-post assessment will also include measures to evaluate transfer effects of the ABM-T to other related relevant cognitive processes in depression such as emotional identification (a novel Emotional Threshold Detection Task, ETDT, based on similar previous paradigms [63]), and interpretation of ambiguous information (a Scrambled sentences task, SST) [64].

**Fig. 1:**
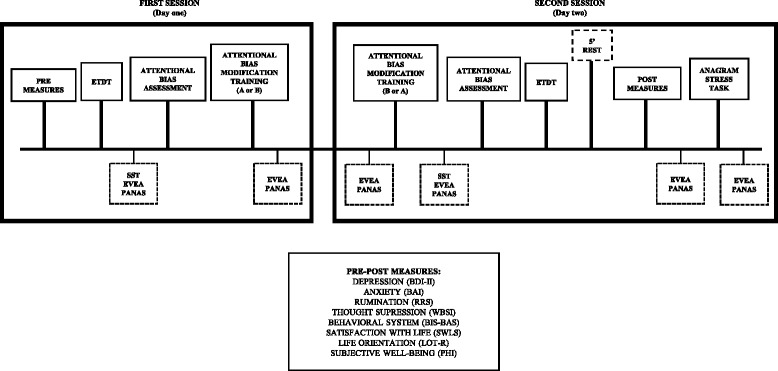
Repeated-measures study procedure

The entire experiment will be conducted over two consecutive days in the laboratory. The ABM-T will be administered during the two sessions, immediately after the attentional pre-assessment with the ABA task (session 1) and immediately before the attentional post-assessment with the ABA (session 2). In session 2, immediately following the ABM-T and the post-assessment with questionnaires, a stress test will be conducted to evaluate the transfer of the ABM-T on stress reactions. At the end of the study, participants will be debriefed by the experimenter. Each of the two sessions will last approximately 80 min (see Fig. 1).

### Participant recruitment and allocation

The sample size will be determined according to power calculations (G*Power; [65]) and based on Cristea et al.’s meta-analysis effect size (*g* = .33) [45]. A total of 32 dysphoric university students, with an estimated age range between 19 and 30 years, will voluntarily participate. All participants must have normal or corrected-to-normal vision via contact-lenses or glasses if required. Students who complete the two-session participation will receive course extra-points or/and a book as a reward for their participation. Participants will be selected based on their scores on Beck Depression Inventory-II - BDI-II [66]. A 13 points cut-off score will be used [67].

**Fig. 2:**
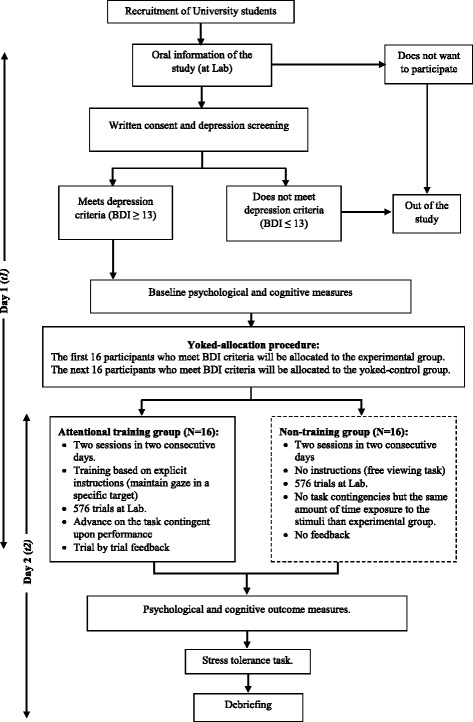
Study flow diagram

The study (see Fig. 2) is a controlled clinical trial with a placebo-control condition and will follow the Standard Protocol Items: Recommendations for Interventional Trials (SPIRIT) Statement for reporting trial protocols (see Additional file 1 for checklist).

### Psychological measures

The BDI-II [66] will be used to evaluate participants’ depressive symptoms. The Spanish version has shown good reliability (α = .87) [68].

The Beck Anxiety Inventory - BAI [69] will be applied to evaluate anxiety symptoms. The reliability of its Spanish version is very good (α = .93) [70].

Ruminative Responses Scale - RRS, [71]. It assesses participants’ rumination cognitive style. The reliability of the Spanish adaptation is very good (α = .93) [72].

The White Bear Suppression Inventory - WBSI, [73] assesses thought suppression tendencies. Its Spanish version has shown a very good reliability (α = .88) [74].

The Behavioral Inhibition System and Behavioral Approach System Scale - BIS/BAS [75] will be used to evaluate general motivational systems. Its reliability is good (α = .73 - .82).

Satisfaction with Life Scale - SWLS [76] evaluates overall life satisfaction. The Spanish adaptation has shown a very good reliability (α = .88) [77].

Life Orientation Test Revised - LOT-R [78] will be used to assess dispositional optimism. Its Spanish version has shown questionable reliability (α = .63) [79].

The Pemberton Happiness Index - PHI [80] is an 11-item self-report scale of hedonic, eudaimonic, and social well-being. Its Spanish version has a very good reliability (α = .92).

The Scale for Mood Assessment - EVEA [81] is a 16-item self-report scale that assesses four different moods (sadness-depression, happiness, anxiety and anger-hostility) with four items each one. The Spanish version has shown very good reliability for each factor (depression (α = .88); happiness (α = .92); anxiety (α = .92); and anger-hostility (α = .93).

The Positive and Negative Affect Schedule - PANAS [82] is a 20-item self-report schedule that assesses the presence of positive and negative affect. The Spanish version [83] has shown a very good reliability both for the Positive Affect factor (α = .92) and for the Negative Affect one (α = .88).

### Attentional Tasks’ stimuli

**Fig. 3:**
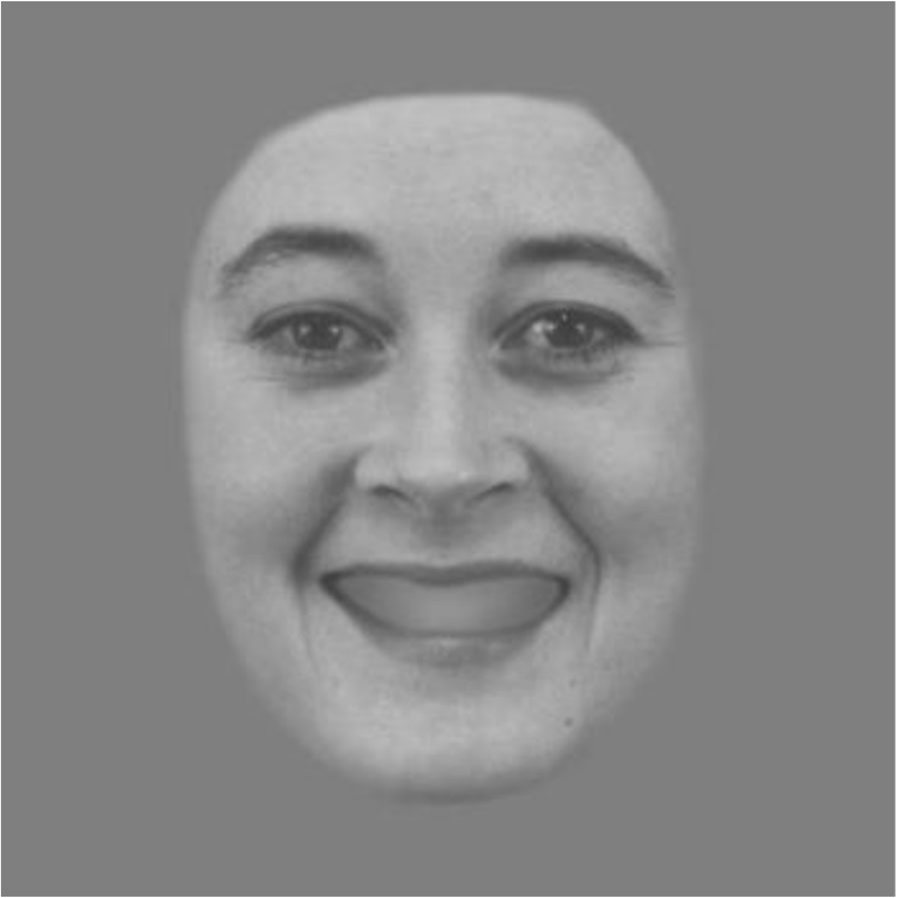
Example of a happy face with teeth blurred using a Gaussian filter. (Model AF14, KDEF)

The Karolinska Institute of Stockholm (Karolinska Directed Emotional Faces - KDEF [84] will be used for the ABA Task. A total of 18 pictures (half men, half women) will be selected from the A series of KDEF displaying two different emotions (i.e., happy and sad). Thus, our procedure, which is similar to that used in other studies one used by [18], will include 36 emotional faces (18 happy, 18 sad) and its corresponding neutral paired face. In the original KDEF database some happy faces have their teeth visible and others not. As teeth visibility artificially attracts viewer’s attention [85], they will be covered by a grey Gaussian-type filter which optimizes gaze patterns towards more informative emotional areas of the face [86] (see Fig. 3). All the pictures will be presented in a frontal view.

In order to avoid possible interference or habituation to the ABA emotional faces, we will use from a different face database for the ABM-T. Twelve pictures (half men, half women) will be selected from the FACES database created [87] at the Max Planck Institute for Human Development (MPIB). As in the ABA task, only two types of emotions will be used (12 happy and 12 sad) plus a set of 12 neutral expressions of the same actors/actress. All the faces will be also presented in a frontal view and the teeth of the happy faces will be also modified following the procedure described above. All pictures will be cropped and edited using Adobe Photoshop CS3. First, faces will be converted to a grey scale and all non-emotional features, such as surrounding parts, will be cropped [88]. All pictures will then be introduced into a grey square background (512 × 512 pixels). To blur the crop procedure, we will shade the outline of the faces into the background. The teeth of the happy pictures will be blurred by using a 10-pixel Gaussian filter. To avoid differences on energy or luminance all the pictures on both tasks, we will equate the contrast energy for assessment and training [86, 89]. Furthermore, a gamma correction will be applied to the 24” BENQ LCD monitor where the stimuli will be displayed. The presentations of the stimuli on the ABA task will be controlled through Tobii Studio software (2.0.6), whereas the stimuli presented during ABM-T will be controlled with an integration of Tobii Studio software (2.0.6) and E-Prime Studio software (2.0.8.22).

### Attentional Bias Assessment Task (ABA)

To assess participants’ attentional bias, we will administer a free viewing task comprising 108 trials [18]. Each trial will begin with a grey screen for 500 ms followed by a white cross fixation displayed at the center of the grey screen. Immediately thereafter, a random white number (1 to 3 and 7 to 9) will replace the cross for 500 ms. To ensure that participants maintain their attention on the fixation cross, we will follow Calvo & Avero's procedure [90] by which participants are asked to read the number out loud as quickly as possible. Afterwards, a pair of competing emotional faces (happy vs. sad; happy vs. neutral; sad vs. neutral) will be displayed for 3500 ms. Participants will be instructed to freely look at the faces, without any constraints, until the beginning of the next trial. Each emotional face will be presented equally often, and its position on the screen (right or left side) will be counterbalanced. In total, the task will be composed of 36 happy vs. sad, 36 happy vs. neutral and 36 sad vs. neutral trials that will appear in random order. The entire task will last about 12 min.

Dependent variables will comprise the latency to first fixation, the duration of first fixation, the number of fixations, and the total fixation time on each emotional face. Furthermore, attentional biases indices will be calculated based on these dependent variables, as done in previous research [18, 54].

### Attentional Bias Modification Training Task (ABM-T)

A wait- for- fixation paradigm will be used in the experimental group to train participants’ attentional patterns. In this paradigm, the progress of each trial depends on the maintenance of participants’ gaze to a previously determined target stimulus during a pre-specified length of time. In our paradigm, participants will be instructed to maintain their gaze on the most positive face of the pair (e.g., in the neutral vs. sad trials participants must hold their gaze at the neutral face; in the neutral vs. happy trials participants must hold their gaze at the happy face). Furthermore, to increase participants’ motivation in the task, especially challenging for depressed people [61], the cognitive demand of the task will increase. Whereas in the first set of trials, participants will have to hold their gaze on the target stimulus for 750 ms, in the second set they will have to do so for 1,500 ms to advance to the next trial. Within each block, participants will first perform the low demand condition (750 ms) before doing the high demand one (1500 ms).

The task will be composed of three independent blocks (happy vs. neutral; sad vs. neutral; and happy vs. sad). Each block, consisting of 96 trials, will be administered twice (i.e., one in each of the two training sessions). Both sessions will finish with the happy vs. sad block, as the others blocks will be counterbalanced across subjects. Thus, the entire task will consist on two independent sessions (two consecutive days) with 288 trials each (576 trials in total). Faces will appear equally often, and their position on the screen will be counterbalanced. Also, to reduce habituation and to foster adherence, we will randomly distribute the position of the faces along the horizontal or vertical axis of the screen.

**Fig. 4:**
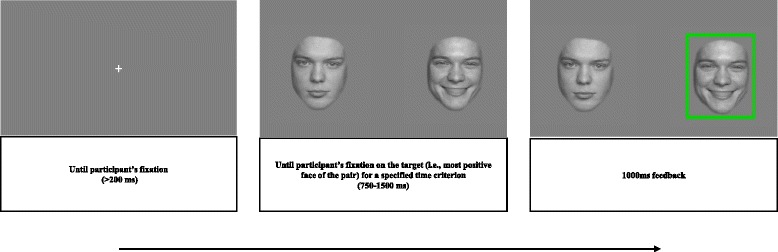
Attentional Bias Modification Training Task. Example of a happy-neutral condition trial. (Model 066, FACES)

In every block and condition, each trial will begin with a grey screen for 500 ms, followed by a white fixation cross appearing at the center of the screen until the participants fixate their gaze for 200 ms on it [13, 15]. After this initial required fixation, two faces will be simultaneously displayed on the screen until the required fixation on the target (i.e., 750 ms or 1,500 ms) is detected by the eye-tracker. As an immediate consequence of this fixation, a green square will be displayed on the face where the fixation has taken place (e.g., a neutral face in a neutral-sad trial; a happy face in a neutral-happy trial), thus providing immediate feedback to the participant. Each training session will last approximately 30 min (see Fig. 4).

### Control group in the attentional bias modification training task (yoked-control group)

For participants in the experimental group, the wait-for-fixation ABM-T paradigm will imply that each trial ends once the participant has spent a given amount of time looking at an area of interest (e.g., a happy face). Thus, in the experimental group, the duration of each trial is contingent upon the time each participant devotes to the target stimulus. An ideal control condition would be one in which participants in that condition would be exposed to the same amount of stimuli and the same total time of exposure.

To achieve this goal, we will use a yoked control group [91]. Participants in the experimental group will be the first ones to participate in the study, and we will measure the average total time they spend on each trial. Control participants will be exposed (“yoked”) to the same amount of time as participants in the experimental group, but they will not receive any type of explicit instruction or feedback. In carrying out this novel procedure in the context of ABM, we will ensure that participants in the experimental and control groups differ only in one way: the experimental group will experience a contingency between their gaze behavior and feedback, whereas the control group will not.

### Eye-tracker device

The location and movements of the participants’ eyes will be recorded and measured with a Tobii tx-120 infrared eye-tracker system with a 120 Hz frequency (approximately, coordinates were recorded every 8.35 ms). The distance between the eye tracker and the participants’ eyes will be 60 cm, controlled by an anatomic chair maintaining the participants’ head in a comfortable, but stable, position. Moreover, a five-point calibration will be done before starting each attentional task. These procedures will occur in a sound-proof room.

### Emotional Threshold Detection Task (ETDT)

To evaluate transfer of training effects following ABM-T, we will administer the ETDT task to assess participants’ thresholds to detect changes in the facial expression of emotions [63]. A total of 408 synthetic faces from two different models will be used (one man, one woman). The models will display two emotions related to training (i.e., happiness and sadness) as well as two emotions unrelated to training (i.e., disgust and anger). These faces have been created by the FACSGen software [92, 93] which controls the emotional intensity displayed by each face. A video-clip will be created for each emotion. Each video-clip will consist of 51 synthetic faces varying their emotional intensity from 0% (neutral state) to 100% (high emotionality) as determined by FACSGen criteria.

In this task participants will be presented with 16 morphing video-clips (4 Emotions × 2 Gender × 2 Condition) and will be asked to press a button when the given emotion emerges from a neutral expression (appearance of emotion condition) or when a given emotion disappears and morphs into a neutral expression (vanishing emotion condition). Each trial will begin with the trial instruction (e.g., “*Indicate when the face ceased to be sad*”), then the morphing video-clip will appear in the center of the screen for 5.100 ms or until participants’ response. A measure of emotional threshold detection will be derived by the participants’ reaction times. Overall, this task may allow detection of subtle changes in the identification of a given emotion as a result of the ABM training.

### Scrambled Sentence Task (SST)

This is an experimental task designed to evaluate interpretation biases [64]. It consists of 20 scrambled sentences of 6-words each. Participants’ task is to unscramble the sentences by using only 5 words. Each sentence can be unscrambled in a positive or negative manner (e.g., equal am others I inferior to). Cognitive load (i.e., remember a 6-digit number) will be used to deplete working memory resources and enable the appearance of negative schemas. Participants will be instructed to unscramble as many sentences as possible in 2.5 min. A measure of interpretation bias will be derived by computing the proportion of negatively unscrambled sentence divided by the total of unscrambled sentences [64].

### Anagram Stress Task (AST)

To assess stress tolerance and changes in emotional vulnerability after attentional training, we will administer an adaptation of the anagram stress task procedure [31]. Participants will be informed that their cognitive performance will be assessed by anagrams’ resolution. The task will comprise 40 anagrams (20 difficult but soluble anagrams, and 20 irresoluble anagrams), and each anagram will appear on the screen one at a time. Participants will be instructed to solve as many anagrams as possible in a 3-min period. At the end of task, a false feedback slide (*“Your performance has been much worse than the average”*) will appear displayed, and participants’ mood will be assessed. At the end of the experiment participants will be fully debriefed by the experimenter about the purpose of the procedure.

### Statistical analysis plan

To analyze the effects of ABM-T on attentional patterns after the attentional training between both groups, we will conduct a series of 2 (Group: Experimental, Control) × 2 (Time: pre-training, post-training) × 3 (Trial: happy vs. neutral, sad vs. neutral, happy vs. sad) mixed ANOVAs. When significant interactions are found, we will conduct a 2 (Group: Experimental, Control) × 2 (Time: pre-training, post-training) ANOVAs for each type of trial.

Furthermore, 2 (Group: Experimental, Control) × 2 (Time: pre-training, post-training) mixed ANOVAs will be carried out to evaluate changes on the different psychological measures. The same 2 (Group: Experimental, Control) × 2 (Time: pre-AST, post-AST) ANOVAs will be performed to assess differences between groups on stress tolerance and vulnerability to the AST. For all analyses, α level will be set at 0.05.

## Discussion

Although initial positive results of ABM led some authors to propose it as an alternative treatment for emotional disorders [35, 94], some discordant voices [95] and subsequent meta-analyses [45, 46] have reduced the enthusiasm of those previous claims. As often happens in the history of science, there is a time-lag effect in the publication of novel findings by which earlier studies, for a variety of reasons [45, 96], show greater effect sizes than later do replications.

The rationale of our ABM proposal is to enhance participants’ maintenance of attention to positive faces. For pairs of sad-neutral faces, the individual will have to search for the less negative and to keep his/her gaze for a given time in that stimulus. The attentional difficulties in depression are, in part, related to excessive engagement with negative information, and, therefore, procedures directly aimed at reducing excessive attentional maintenance and difficulties disengaging attention from negative sources of information are conceptually appropriate. On the other hand, for pairs of neutral-happy faces, participants’ task will be to identify the positive emotion and to keep their attention on it. For this experimental condition, there is also a consistent theoretical argument. In an extensive review of the literature on neuroimaging studies, [11] the authors showed that depression is characterized not only by an attentional bias toward negative stimuli, but also by filtering out of positive stimuli. Therefore, in the case of depression it is conceptually consistent to work simultaneously with interventions addressed at modifying both types of biases. In order to target impairments due to this co-occurring double bias, we would combine the re-training of both attention patterns (i.e., in trials of happy-sad faces, participants must both avoid sustaining their attention on negative information as well as to sustain it on positive information).

The presentation of different conditions of processing (i.e., detect and maintain attention on the less negative picture in sad-neutral trials; detect and maintain attention on the most positive picture in happy-neutral, happy-negative trials) requires a degree of top-down attentional control implementation that participants may learn to extend in real demanding situations, characterized by dynamically changing emotional contexts. The guidance of attention processing in our procedure is achieved by the introduction of external contingencies [15, 57, 97] to provide performance feedback under increasingly demanding conditions may foster adherence and bolster motivation.

The proposed study aims to rectify several limitations of previous ABM designs while opening a new strategy, based in training ocular movements, to modify attentional patterns. With the methodological and conceptual improvements described earlier (i.e., trial-by-trial feedback, use of different tasks to measure attentional bias and to do the ABM, use of a yoked-group design to control for the time exposure to the emotional stimuli in the control group, and use of a stress-test to measure transfer of the training to a different task), it is expected that some limitations found in previous studies can be overcome. Furthermore, the use of the new ABM in a sample of dysphoric participants will allow us to test if training visual selective attention using eye-tracking methodology could be a promising venue for future ABM procedures more solidly grounded on current theories of depression.

### Trial status

The trial was retrospectively registered on 28 July 2016, with ClinicalTrial.gov NCT02847793. The study is actively recruiting participants and ongoing.

## Additional file

Additional file 1: The SPIRIT checklist for this study is included. (DOC 122 kb)

## Abbreviations

ABA: Attentional bias assessment
ABM: Attention bias modification
ABM-T: Attentional bias modification training
AST: Anagram stress task
BAI: Beck anxiety inventory
BDI-II: Beck depression inventory-II
BIS/BAS: Behavioral inhibition system/behavioral approach system
CBM: Cognitive bias modification
ETDT: Emotional threshold detection task
EVEA: Scale for mood assessment
KDEF: Karolinska directed emotional faces
LOT-R: Life orientation test-revised
MPIB: Max Planck Institute for human development
PANAS: Positive and negative affect schedule
PHI: Pemberton happiness index
RRS: Ruminative responses scale
SST: Scrambled sentences task
SWLS: Satisfaction with life scale
WBSI: White bear suppression inventory
